# The Hematological and Biochemical Manifestations of Cutaneous Leishmaniasis in a Shih Tzu-Terrier Dog with Severe Infection: A Case Report

**DOI:** 10.18502/ijpa.v15i3.4213

**Published:** 2020

**Authors:** Mahmooud AHMADI-HAMEDANI, Hediyeh HOSSEINPOUR, Hesamodin ESKAFIAN, Shayan DAVARPANAH

**Affiliations:** 1. Department of Clinical Sciences, Faculty of Veterinary Medicine, Semnan University, Semnan, Iran; 2. Veterinary Medicine, Semnan University, Semnan, Iran

**Keywords:** Canine leishmaniasis, Pathology, Cutaneous, *Leishmania*

## Abstract

Cutaneous involvement in canine leishmaniasis, caused by *Leishmania infantum*, is the most frequent clinical manifestation of the zoonotic infectious disease. A 4-month-old female Shih Tzu-terrier dog with significant weight loss and depression and chronic erosive skin lesions around eyes and the area above the nose was presented to the Semnan University Veterinary Hospital teaching, Semnan, Iran. The main clinicopathological findings included marked leukocytosis, neutrophilia, left shift, monocytosis, mild hypoproteinemia, and hypoalbuminemia. The diagnosis of leishmaniasis was performed based on the presence of a large number of *Leishmania* amastigotes in skin Fine Needle Aspiration (FNA). The dog was euthanized and sent to the autopsy department for further investigation.

## Introduction

Canine cutaneous leishmaniasis is an infectious zoonotic disease transmitted by female sand fly’s bite of the genus *Phlebotomus* and *Lutzomiya* from the Old World and the New World, respectively (1). The main route of transmission of the disease to humans and dogs are promastigotes (the infectious stages). There are other routes of transmission including vertical (2, 3), venereal (3), infected blood (4, 5) and the direct transmission (6, 7). The reservoir hosts of leishmaniasis are stray dogs, wild canids, and carnivores such as jackals and foxes in some areas of Iran (8).

Clinical symptoms of leishmaniasis can be varied from focal cutaneous to disseminated visceral lesions. The most prevalent form of leishmaniasis is the cutaneous type that is divided into two subtypes included dry (urban) and humid (suburban) wound. It is estimated that 12–14 million types of this illness exist in the world (9), 70%–75% of cutaneous leishmaniasis has been reported from Afghanistan, Algeria, Columbia, Brazil, Iran, Syria, Ethiopia, North Sudan, Costa Rica, and Peru (10). The prevalence of cutaneous leishmaniasis in various parts of Iran is variable from 1.8% to 38% (11).

Two common forms of the disease have been reported from Iran; visceral (urban) and cutaneous (suburban); in urban leishmaniasis or anthroponotic cutaneous leishmaniasis (ACL), the cause of the illness is *L. Tropica*, its vector is *Ph. sergenti*. The urban-type of this disease is reported from almost 14 centers in eight cities all around Iran; these reports are mostly from metropolises, like Tehran, Mashhad, Neyshabour, Shiraz, Kerman, and Bam (10).

Zoonotic cutaneous leishmaniasis is reported from almost all of the suburban parts of 17 cities all over the country (12). In Iran, *L. gerbil* and *L. turanica* are diagnosed in rodents (13). Renal failure is a common finding in canine leishmaniasis, which has been associated with thrombosis or disseminated intravascular coagulation (14). Liver illness is less common in canine leishmaniasis (15).

## Case Presentations

**Fig. 1:**
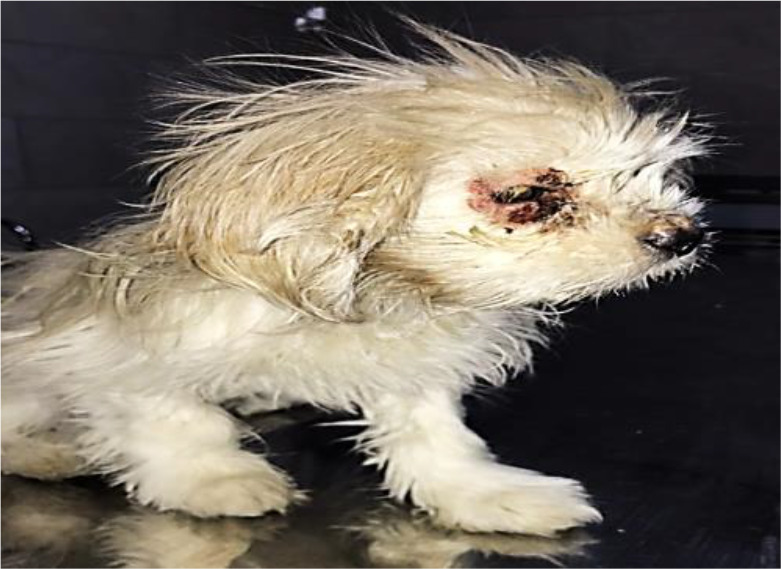
Cutaneous lesions around the eye of the animal

A 4-month-old female Shih Tzu-terrier dog with significant weight loss and depression (Fig. 1) was admitted to the Veterinary Hospital of Semnan University of Iran.

All stages performed in this study approved by the Iranian animal ethics framework under the supervision of the Iranian Society for the Prevention of Cruelty to Animals and Semnan University Research Council (Ethical Code: EC976). The owner of the studied dog stated their written consent.

The dog has received treatment with antibiotics for 3 weeks in other veterinary clinics and had not responded to antibiotic therapy at all, the condition of her wounds got worst by the pass of time.

Lymphadenopathy and chronic erosive skin lesions around eyes and the area above the nose were found on physical examination papule. FNA was taken from cutaneous lesions and impression smear was prepared for direct microscopy diagnosis. Blood samples were collected from the jugular vein for Complete Blood Count (CBC) and Plasma was obtained for determination of biochemical parameters.

**Fig. 2:**
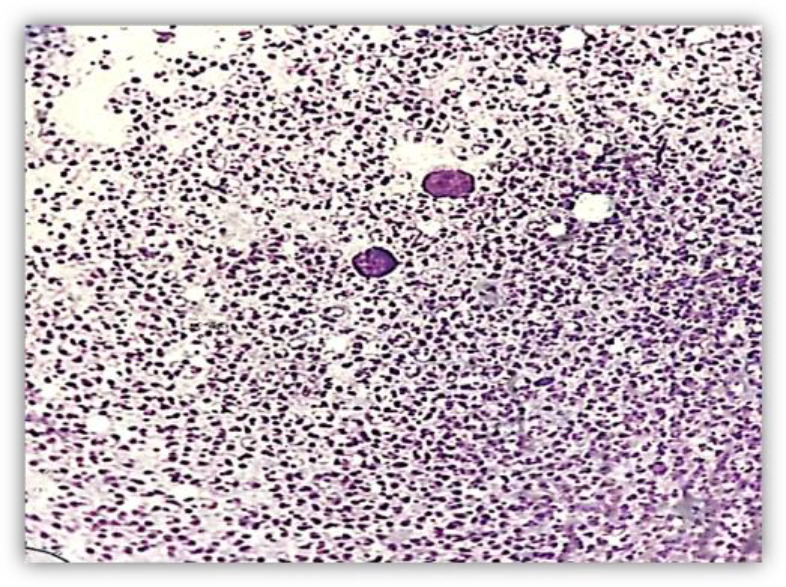
Giemsa-stained FNA smear prepared from skin. A large number of *Leishmania* amastigotes are visible (Original picture)

**Fig. 3:**
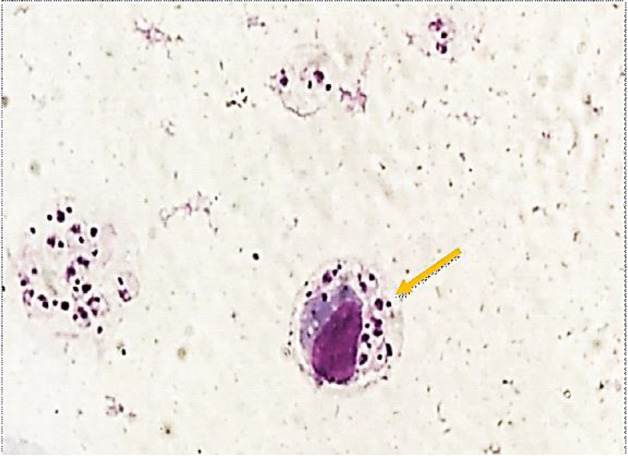
Giemsa-stained FNA smear prepared from skin. Intracellular *Leishmania* amastigotes in a macrophages are visible (Original picture)

In microscopy evaluation of smears were obtained from FNA samples and stained with Giemsa, were found a large number of *Leishmania* amastigotes inside and outside of macrophages (Figs. 2, 3).

Marked leukocytosis (WBC 28,900 μL), neutrophilia (segmented 22,253/ μL), left shift (bands 867/μL) and monocytosis (monocytes 2,601/μL) were detected from hematological examination. Mild microcytic hypochromic anemia was also reported by the laboratory (Table 1). The most important of biochemical changes in the plasma were mild hypoproteinemia, and hypoalbuminemia (Table 2).

**Table 1:** Hematological findings in Shih Tzu-terrier dog with Leishmaniasis

***Hematological Tests***	***Measured***	***Normal range***
WBC ×10^3^/μL	28900	5050–16760
RBC ×10^6^/μL	6.43	5.65–8.87
HGB g/dL	11.1	13.1–20.5
HCT %	39.3	37.3–61.7
MCV fL	61.1	61.6–73.5
MCH Pg	17.3	21.2–25.9
MCHC g/dL	28.2	32–37.9
PLT ×10^3^/μL	444	148–484
Neut	22253	2950–11640
Lym	3179	1050–5100
Mon	2601	160–1120
Eos	0	60–1230
Band	867	0–300
RDW %	16.5	13.6–21.7
PCT %	0.34	0.14–0.46
MPV fl	7.6	8.7–13.2
PDW %	16.4	9.1–19.4

**Table 2:** Serum biochemical profiles in Shih Tzu-terrier dog with Leishmaniasis

***Biochemistry Tests***	***Measured***	***Normal range***
ALP U/L	201.22	35–280
AST U/L	28.25	16–40
ALT U/L	28.68	10–120
Tp mg/dL	4.12	5.4 – 7.4
Albumin mg/dL	1.65	2.6 – 3.3
Glob mg/dL	2.47	2.7–4.4
A/G	0.66	0.59–1.1

## Discussion

A few studies have shown hematological and serum biochemical changes occurred by canine Leishmaniasis (16, 17), therefore the present study investigated hematological and serum biochemical alterations in a Shih Tzu-terrier dog naturally infected by cutaneous leishmaniasis. In this case, with specific cutaneous lesions on the animal’s face, the diagnosis of leishmaniasis was confirmed by direct observation of a large number of *Leishmania* amastigotes inside and outside of macrophages in light microscopy of the stained smears prepared from FNA samples collected from cutaneous lesions, which is referred to as the gold standard for the diagnosis of leishmaniasis (18).

Mohebali et al (19) indicated that canine visceral leishmaniasis (CVL) is transmitted in endemic areas of Iran by the potential main reservoir of parasites, dogs, and other carnivores such as cats and rodents play an important role in transmitting the infection. The sylvatic transmission cycle of VL occurs in the endemic area of Iran by wild dogs (20).

Cytology is strongly suggested for the diagnosis of canine cutaneous leishmaniasis when lesions are accessible to FNA (lymphadenopathy, nodular lesions, joint swelling). In cases where cytology is not a determinant, the diagnosis should be made by histology/immunohistochemistry or PCR on surgical biopsies (21).

One of the common hematological findings of canine leishmaniasis is anemia which in the present study is documented the presence of anemia in this case with severe cutaneous leishmaniasis. This anemia manifests as a normocytic normochromic form. Several factors have been suggested for the onset anemia in canine leishmaniasis, including extravascular hemolytic anemia due to phagocytosis of RBC by the enlarged spleen and liver caused by inflammatory response (22) or impaired erythrocyte membrane fragility in leishmaniasis (23), anemia of chronic diseases (24) and erythropoietic linages hypoplasia (25).

It seems neutrophilia, that is mostly caused leukocytosis, has been related to the cutaneous lesions resulted by the secondary infection (26). The impaired Leukogram such as leukocytosis, neutrophilia, left shift and monocytosis was obtained from this case, are the most remarkable characteristics in canine leishmaniasis (27, 28). Secondary infection of the cutaneous lesions leads to these changes in the leukogram (26).

In this study, the *Leishmania*-infected dog had low concentrations of serum Tp and albumin. The manifestation of Tp reduction in canine cutaneous leishmaniasis is completely contradictory with canine visceral leishmaniasis and requires more attention (29, 30). It can be admitted that the reduction of serum Tp has been caused by severe skin lesions and excessive loss of appetite in the case.

## Conclusion

The severe form of cutaneous leishmaniasis in the presented case could cause marked clinicopathological manifestations. These changes include anemia, leukocytosis, neutrophilia with left shift, monocytosis and hypoproteinemia, and hypoalbuminemia.
